# River blindness: reducing the prevalence of clinical disease

**Published:** 2018-06-03

**Authors:** Charles Mackenzie, Martin Kollmann, Sabine Specht, Yao Sodhalon

**Affiliations:** 1 Senior Technical Adviser: Task Force for Global Health, Atlanta, USA.; 2 Senior Advisor for Neglected Tropical Diseases: CBM, Nairobi, Kenya.; 3 Senior Scientist: Institute for Laboratory Animal Science, University of Zurich, Switzerland.; 4 Director: Mectizan Donation Program, Georgia, USA.

**It may be time to widen the focus of onchocerciasis programmes to include the prevention and treatment of clinical disease of the eyes and skin.**

**Figure:**
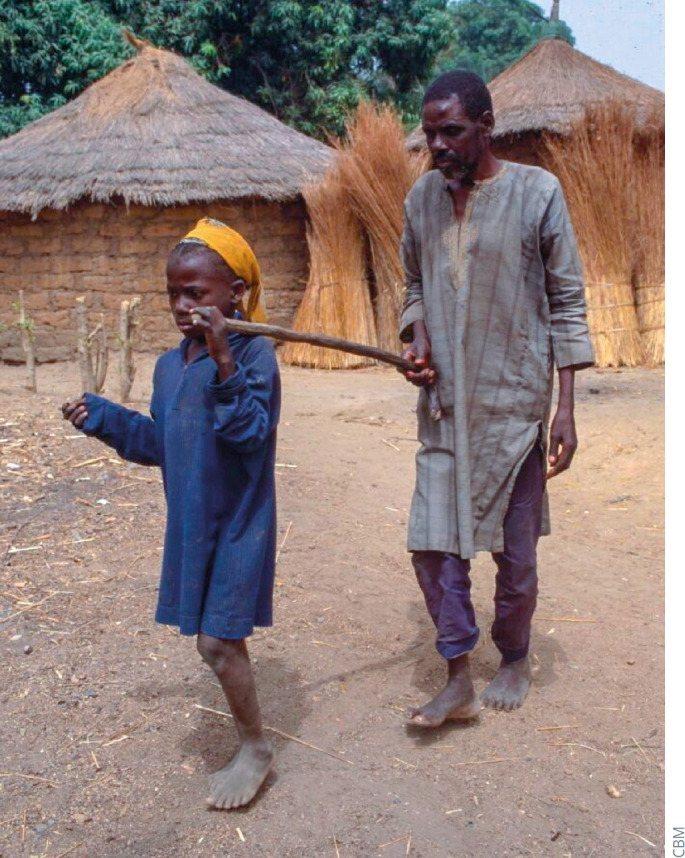
A boy guides a man who has river blindness. WEST AFRICA

The once well-known - and disturbing - image of a person with river blindness (onchocerciasis) is now thankfully much less common, thanks to successful ivermectin distribution programmes that have reduced the prevalence of *Onchocerca volvulus* infection over the past 30 years.^1^

However, as we celebrate this remarkable success, we must not forget those people who are still affected by clinical onchocerciasis (onchocercal eye and skin disease). There is a general perception that onchocerciasis is now much less of a public health problem than in past decades, and that new cases of onchocercal disease are few. This assumption needs to be backed up by solid research, especially in countries that are highly endemic and/or where the distribution of ivermectin has not been regularly achieved due to violent conflict. These countries include South Sudan and the Democratic Republic of Congo.

Surveys of onchocercal eye disease, which were common some 30 years ago, are rarely conducted these days.^2^ A major factor for this is the current focus on elimination of transmission rather than prevention of disease.^3^

Unlike the global lymphatic filariasis programme, the global onchocerciasis programme has not had a strong individual patient care component in recent years. This is understandable, in part, because ivermectin is also used to reduce the clinical symptoms and signs of onchocercal disease. In addition, the expertise (ophthalmology and dermatology) needed for clinical assessment has not been readily available to national onchocerciasis programmes. Approaches to the care of those with onchocerciasis are listed in Table 1.

However, perhaps the most important unknown is the lack of reliable figures as to how many people are suffering from onchocercal eye and skin disease. This information is needed to provide treatment and care for those affected by both existing and new disease. Knowing the true reduction in eye and skin disease will allow us to accurately document the success that has been achieved and identify remaining any gaps that need attention. It should also encourage further support for those who have irreversibly lost vision or suffer from the severe forms of onchocercal skin disease.

As neglected tropical diseases gain ever more prominence in the context of Universal Health Care, we need to celebrate the successes in reducing the devastating effects of all diseases including onchocerciasis. Clinical onchocerciasis results in significant disability for the affected individual, which also impacts families and communities. We must actively look for remaining cases - both old and new - as is done with other disability- and stigma-inducing diseases such as lymphatic filariasis and leprosy. We should remember that the Global Program for Onchocerciasis Control was begun because there were patients that needed treatment and support. The ongoing challenge now is finding out how many people are still suffering from clinical onchocerciasis and its consequences - and what is being done to help them.

**Table 1:** Approaches to providing care for people with onchocerciasis

	Approach	Target community	Comments
**A**	Community drug distribution (PCT)	Whole endemic community	Will eventually reduce/eliminate incidence of new disease
**B**	Individual patient treatment/management	Those presenting with specific onchocercal symptoms	Includes ivermectin as well as specialised care for eyes and skin
**C**	Rehabilitation and care	Those permanently blind and their families	Includes links to associations for the blind, self-care groups, etc.
